# The eagle jugular syndrome

**DOI:** 10.1186/s12883-019-1572-3

**Published:** 2019-12-21

**Authors:** Paolo Zamboni, Alba Scerrati, Erica Menegatti, Roberto Galeotti, Marcello Lapparelli, Luca Traina, Mirko Tessari, Andrea Ciorba, Pasquale De Bonis, Stefano Pelucchi

**Affiliations:** 1 Vascular Diseases Center, Ferrara University Hospital, Ferrara, Italy; 2 Neurosurgery Unit, Ferrara University Hospital, Ferrara, Italy; 3 Interventional Radiology Unit, Ferrara University Hospital, Ferrara, Italy; 4 Vascular Surgery Unit, Ferrara University Hospital, Ferrara, Italy; 5 ENT Unit, Ferrara University Hospital, Ferrara, Italy

**Keywords:** Eagle syndrome, Jugular compression, Elongated styloid process, Perimesencephalic subarachnoid haemorrhage

## Abstract

**Background:**

The elongation of the styloid process is historically associated with two variants of the Eagle syndrome. The classic one, mainly characterized by pain and dysphagia, and the carotid variant characterized by pain and sometimes by cerebral ischemia. We observed a further variant characterized by a styloid elongation coursing adjacent to the transverse process of C1, causing significant compression of the internal jugular vein.

**Methods:**

We reviewed all the cases of Eagle syndrome, including the jugular variant, admitted in our Hospital in the last six years.

We compared symptomatology, associated comorbidities and imaging. Data were statistically analyzed.

**Results:**

Overall 23 patients were admitted to the Hospital for symptomatic elongation of the styloid process, 11 male and 12 females. The jugular variant of the Eagle syndrome is clinically delineated by significant differences, as compared to the classic variant and carotid variants. Headache was the more prominent symptom (*p* < .009) as well as a documented peri-mesencephalic hemorrhage was the more significant comorbidity (*p* < .0003). The group classic-carotid variant was characterized by ipsilateral pain respect to the jugular variant (p < .0003). CT angiography with venous phase extended to the neck veins and imaging reconstruction is highly recommended as imaging technique, complemented by color-Doppler ultrasound.

**Conclusions:**

The elongation of the styloid process may have different paths which creates compression on the surrounding anatomical structures. There may be a possible association of jugular impingement by an elongated styloid process with symptoms.

**Trial registration:**

Protocol n°45–2013.

## Background

In 1937, Eagle proposed two syndromes associated with an elongated styloid process, respectively a classic syndrome and a carotid artery syndrome. The former seems facilitated by scar tissue developed after a tonsillectomy which stretches the nerve endings in this area, mainly causing pain, dysphagia and otalgia [1–7]. The latter is thought a consequence of an elongated styloid process impinging upon the carotid artery and associated nerve endings, causing pain and sometimes facilitating vascular complications and cerebral ischemia [8–11]. In both syndromes, the length of the styloid process was thought to be the major causal factor. In addition, it has also been observed a variant of the Eagle syndrome, where an elongated styloid process is coursing adjacent to the transverse process of C1 [12–14]. Often, in this anatomical situation, a significant compression of the internal jugular vein (IJV) can be observed. However, few anecdotal reports are available regarding symptoms and clinical presentation of such jugular vein bone nutcracker [12–14]. In some articles the symptoms seem similar to the classic syndrome, whereas others report unsuspected symptoms and complications such as intracranial hypertension and cerebral hemorrhage [13–16].

Despite the heterogeneity of the symptomatology of the Eagle clinical picture [16], we hypothesized that the jugular variant could be characterized by a more specific clinical picture linked with the venous obstruction.

Aim of the present study is to compare the jugular variant of the Eagle syndrome with the other more common presentations, from a clinical, imaging, and hemodynamic point of view.

## Methods

An extensive review of cases of symptomatic elongated styloid process who were admitted and eventually treated in our University Hospital in the last 6 years was performed.

Institutional review board approval was obtained for this retrospective analysis. A detailed medical history, clinical examination, 3D head CT scan with contrast were undertaken in all patients. In addition, cases with carotid or jugular involvement underwent a hemodynamic study by the means of color Doppler ultrasound (CDU).

Patients affected by classic, symptomatic Eagle’s syndrome and surgical treated at the ENT Department of the University Hospital of Ferrara have been selected for this study. Physical examination included careful palpation of the tonsillar fossa, lateral pharyngeal wall, chewing muscles and above mandibular angle in an attempt to determine the patient’s discomfort. The diagnostic workup included an imaging assessment (orthopantomography, neck CT scans or neck MRI scans). In addition, we reviewed patients with symptomatic carotid variant of the Eagle’s syndrome who were investigated and treated at the vascular surgery unit, by the means of CDU and 3D angio CT scan.

Finally, patients with radiological and/or CDU evidence of an elongated styloid process of extracranial jugular or carotid compression above the carotid bifurcation were respectively collected from the Neurosurgery unit and Vascular Diseases Center.

Patients clinical history was collected, they underwent a CT angio, angio-MRI, CDU of extracranial vessels and a clinical-neurological evaluation. All CTAs were reviewed and eventually reconstructed by the means of proper softwares by three Authors. Surgery was proposed to patients with medically untreatable neurological symptoms. Indeed, the prolonged and sustained intracranial hypertension due to a reduced cerebral venous outflow and not manageable by medical therapy (such as acetazolamid) can cause unbearable headache, papilledema and progressive cognitive impairment.

### DUS assessment of brain haemodynamics

The doppler ultra sound (DUS) protocol, previously described [17, 18] is aimed to assess respectively the brain inflow, the brain outflow, and the venous collateral flow index. The DUS assessment of the cerebral inflow consists in sampling the common carotid artery, the external and the internal carotid artery and the vertebral artery (VA). Parameters of time average velocity (TAV), cross sectional area (CSA), and flow rate were assessed according to reporting standard. For the assessment of the outflow the internal jugular veins (IJVs) were insonated at classic J3 level (upper part of the neck just below the jugular foramen), J2 (at the level of the mid thyroid gland), and J1 level (outlet with the subclavian vein), as well as the vertebral vein (VV) at V2 level.

### Statistical analysis

Data are given as mean and standard deviation. The association between the presence of an elongated styloid process and symptoms and/or cerebral comorbidities in the classic variant group in comparison with the jugular variant group were analyzed by the means of two-sided Fisher exact test. *P* value < 0.05 were considered significant.

## Results

Overall, patients assessed in our University Hospital in the last 6 years for Eagle syndrome were 23 They were 11 males and 12 females. They were subdivided into 3 groups respectively 14 patients with the classic, 1 patient with the carotid, and 8 patients with the jugular variants. The only patient with the carotid variant was a 48 y.o. female, who was admitted for carotid dissection complicated by thrombosis. She spontaneously recovered symptoms from cerebral ischemia. She was excluded from subsequent comparative evaluations.

Clinical symptomatology and associated neurological comorbidities are given in Table 1. The jugular variant of the Eagle syndrome is clinically delineated by significant differences, as compared to the classic and carotid variant. Headache was the more prominent symptom (*p* < .009) as well as a documented peri-mesencephalic hemorrhage was the more significant comorbidity (*p* < .0003). The group classic variant was characterized by ipsilateral pain respect to the jugular variant (p < .0003).

**Table 1:** Patients population demographics and clinical presentation of symptomatic Eagle syndrome subdivided according to the respective clinical variant

SYMPTOMS AND ASSOCIATED COMORBIDITIES	CLASSIC SYNDROME	CAROTID VARIANT	JUGULAR VARIANT	RR 95% CI	P.
History of tonsillectomy	1/14	1/1	0/8	N/A	.43
Odynophagia	1/14	0/1	1/8	0.53 0.03–7.44	.58
Dysphagia	4/14	0/1	0/8	N/A	.15
Ipsilateral pain (present/absent)	11/14	1/1	0/8	N/A	.0003
Stabling pain	4/14	1/1	1/8	2.67 0.37–19.09	.29
Periorbital pain extension	0/14	0/1	1/8	N/A	.35
Pain at contralateral head rotation	0/14	1/1	0/8	N/A	.65
Dizziness	1/14	1/1	3/8	0.35 0.07–1.71	.21
Otalgia	3/14	0/1	0/8	N/A	.24
Ipsilateral facial edema	0/14	0/1	1/8	N/A	.35
Numbness	0/14	0/1	3/8	N/A	.03
Headache	0/14	1/1	5/8	0.11 0.01–0.76	.009
TIA/Stroke	0/14	1/1	0/8	N/A	.65
Perimesencephalic hemorrhage	0/14	0/1	6/8	N/A	.0003
Multiple sclerosis	0/14	0/1	2/8	N/A	.011
Dilated ventricles-CSF spaces	0/14	0/1	2/8	N/A	.011

On the Eagle jugular group one patient underwent surgery (see illustrative case) and 7 patients were managed conservatively.

14 patients in this series belong to the classic pattern of Eagle Syndrome. The cohort of patients consists of 6 males and 8 females, (aged between 28 and 68 years, mean 48 ± 9 years). All patients were treated surgically under general anesthesia by cervicotomy and received antibiotic prophylaxis. They were usually discharged in one-two days after surgery (informed consent was collected from each patient prior to surgery, according to the Italian laws); no patient presented perioperative and postoperative complications.

A total of 8 patients with radiological and CDU evidence of extracranial jugular compression due to an elongated styloid process was collected from the Neurosurgery department, 5 male 3 female, mean age 64 ± range 50–87 yo.

**Fig. 1:**
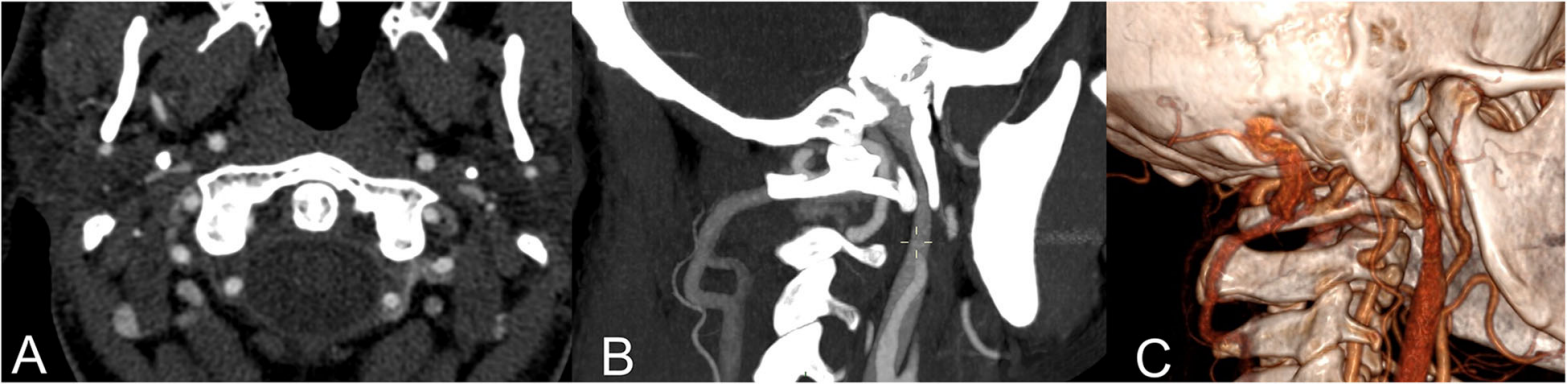
CT angio of the illustrative case showing a right jugular internal vein bone nutcracker between the C1 transverse process and the elongated styloid process. **a**) Axial cut; **b**) Longitudinal reconstruction; **c**) 3D reconstruction

The degree of vein stenosis at CTA were > 90% in all cases (Fig. 1) at C1 level. The corresponding DUS assessment was characterized by flow no-Doppler detectable at J3 level, with increased collateral flow index (> 30% of the inflow). The time-averaged maximum blood flow velocity (TAV) in the contralateral not affected J3 jugular veins ranged between 24 and 38 cm/sec.

Symptoms reported in Table 1 were in part treatable by a conservative approach, especially headache and pain. Numbness and dizziness are still persisting but self-considered not invalidating by the patients. Follow-up is in course. Until now, we proposed surgery to a patient because of the invalidating symptoms which were not controlled by medical treatment, especially headache and facial edema. We are following up the others patients in order to check symptoms deterioration and eventually offering surgery.

### Illustrative case

**Fig. 2:**
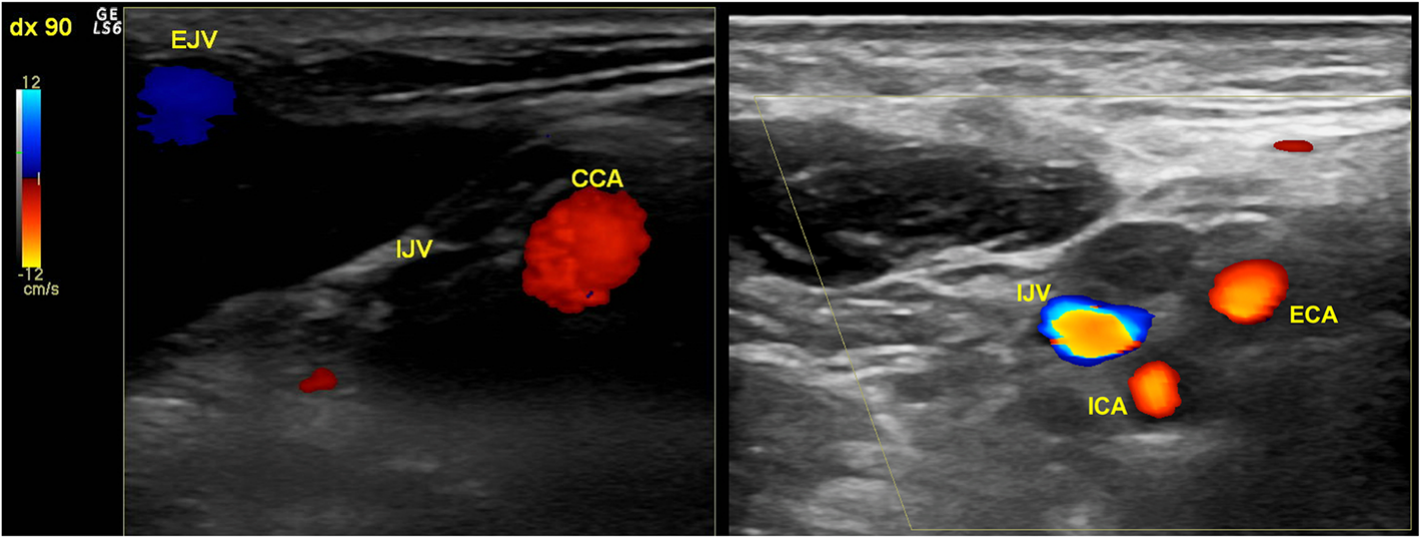
Transverse access CDU of the upper neck of the illustrative case. It is well apparent on the left a collapsed empty internal jugular vein (IJV) without any Doppler flow signal and an enlarged external jugular vein (EJV). CCA: common carotid artery. Right: six months post operatory CDU of the illustrative case, at the level of the carotid bifurcation. A filled and expanded IJV with Doppler flow signal is well apparent. ICA: internal carotid artery. ECA: external carotid artery

**Fig. 3:**
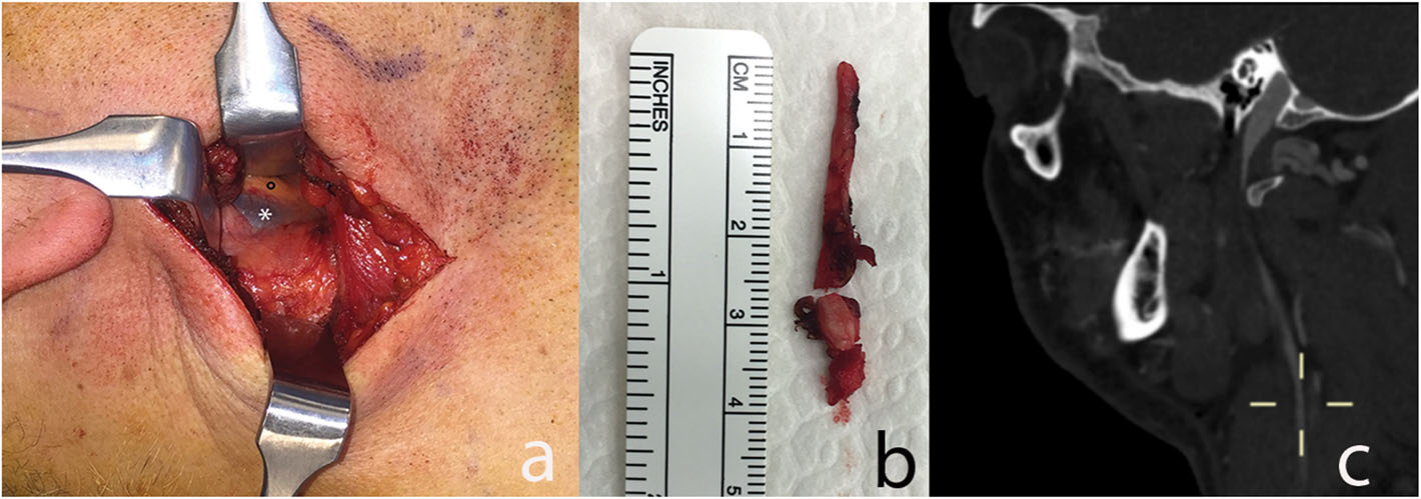
**a**) intraoperative picture of illustrative case, showing the relationship between the elongated styloid process (°) and the internal jugular vein (*); **b**) the elongation of the removed styloid process; **c**) the post-operative CTA showing the obtained decompression of the jugular vein due to the styloid process removal, in the absence of the restoration of its flow. This lead us to perform the following jugular PTA

**Fig. 4:**
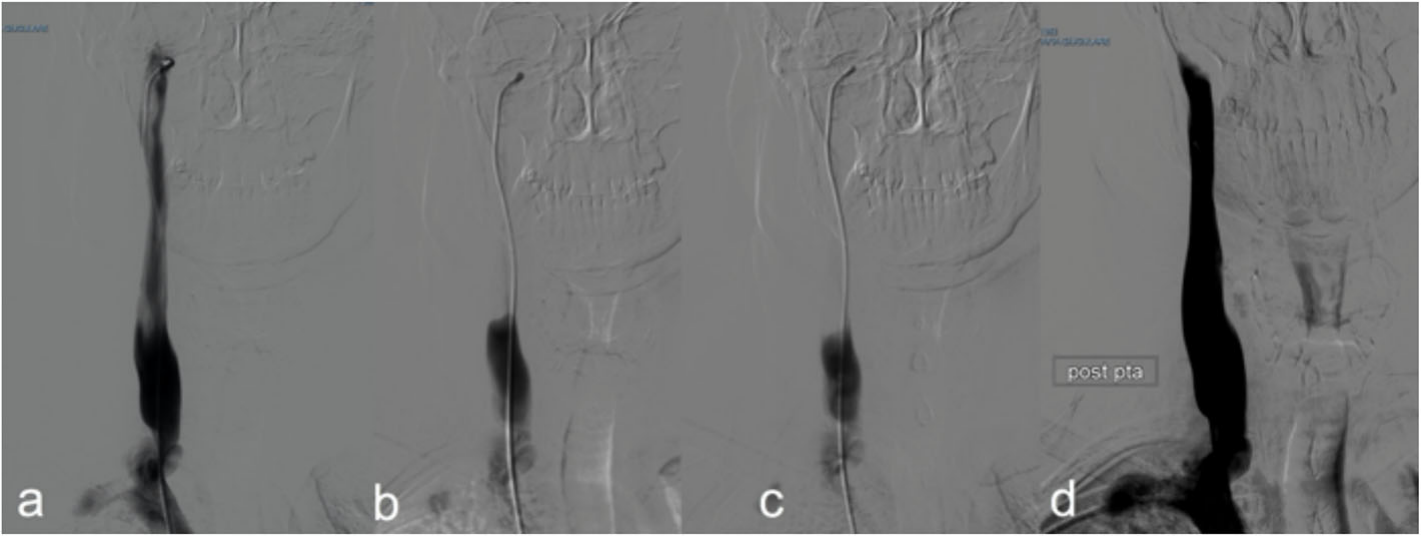
**a**) Catheter venography of the right internal jugular vein showing the significant reduction of the cross sectional area either at the upper level, corresponding to the previous site of compression, or at the lower level, corresponding to a rigid valve apparatus; **b-c**). Delayed clearance of the contrast dye; **d**) Optimal morphological result after balloon angioplasty (PTA), functionally corresponding to a prompt drainage of the contrast dye

One patient with the jugular syndrome presented with a 2 years history of dysesthesia, pain in the right side of the face, soft tissue swelling (especially in the morning) and nuchal and orbital headache resistant to drug treatment. CT angio showed a right jugular compression due to an elongated styloid process (Fig. 1) and a CDU showed compromised cerebral outflow on the right jugular vein, collateral vein circulation on the face and neck and no evidence of intraluminal causes of occlusion (Fig. 2). Because of the persistent symptoms he underwent surgery consisting of a right lateral transcervical approach to the infratemporal fossa and the complete styloidectomy (Fig. 3). The postoperative course was uneventful. A post operative CT angio (Fig.3c) and a CDU failed to demonstrate an expected increase in diameter of the right internal jugular vein, following the surgical decompression. CDU showed flow re-appearance at J3 level, although the flow rate was lower respect to normal values [19]. Facial oedema was slightly reduced but headache did not. In order to obtain a complete restoring of the flow, patients underwent balloon angioplasty either at the level of the J3 residual stenosis or at J1 level, with a satisfactory result [20] (Fig. 4). Symptomatic relief was obtained and patient was stable at a 6 months follow-up.

## Discussion

**Fig. 5:**
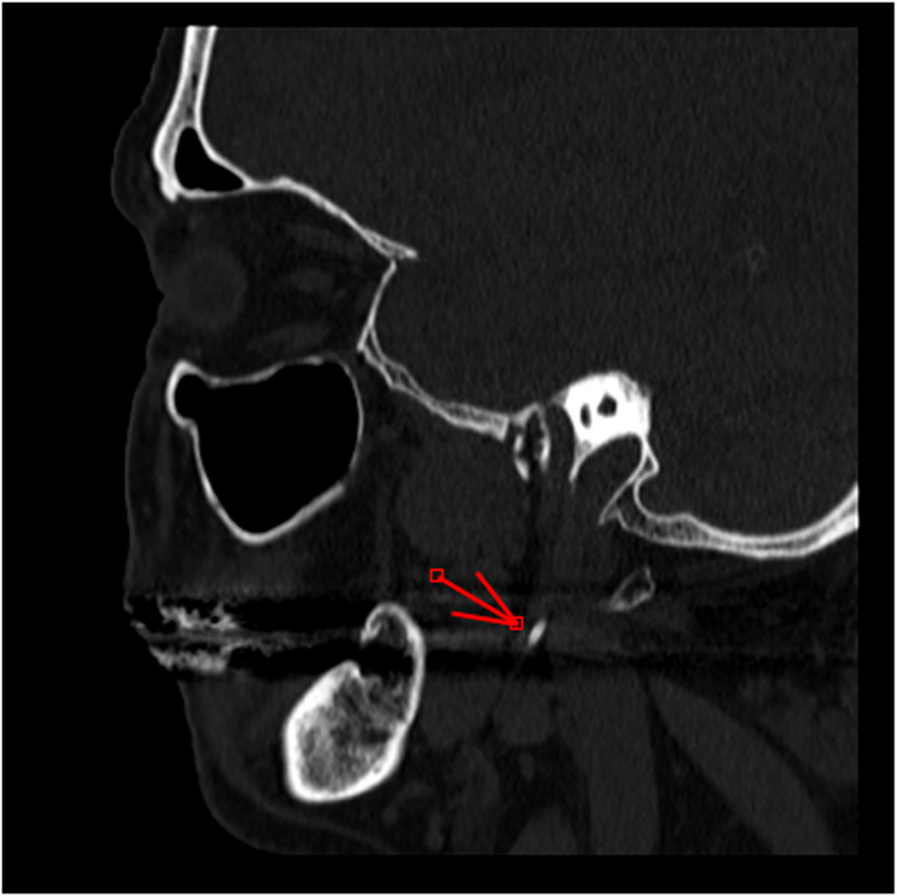
CTA showing a classic Eagle syndrome. The arrow points out the elongated styloid process. The space between this and the transverse process of C1, where the Jugular Vein courses, is wider than the jugular variant

The etiopathogenetic mechanism of the elongation of the styloid process has not been ascertained so far; development abnormalities and/or bone homeostasis alterations have been proposed in order to explain the occurrence of ossification or elongation of the stylohyoid process [1]. Symptoms of the classic Eagle syndrome (Fig.5) have been associated mainly to an intermittent compressive neuropathy involving different branches of cranial nerves, which is often exacerbated by swallowing and yawning [1–7]. These symptoms are caused by a mass effect on cranial nerves eliciting a neuropathic pain which poorly responds to medications. Nowadays the literature tends to support that surgical treatment results in a more definitive treatment and long lasting symptomatic relief [21]. Other symptoms reported in literature are sense of hypopharyngeal foreign body, odynophagia, dysphagia in addition to cervico-facial pain with different irradiations. Cases of Horner’s syndrome are usually explained with the compression of the nerve fibers of sympathetic nervous system [1]. Apart from the classic pattern, also a ‘carotid’ variant has been described, where peculiar symptoms such as arterial dissection, obstruction, transient ischemic attack and cerebral ischemia were found associated [8–11]. It is more difficult to explain cases of Eagle syndrome associated with migraine and headache, which could be more consistently related to the external compression of internal jugular vein (Fig. 1).

Both at surgery and CTA the classic group did not show any involvement of the jugular vein, and this may explain us the lack of headache in our classic group (Table 1).

Clinically relevant extrinsic compression on the extracranial internal jugular vein (IJV) due to an elongated styloid process has been rarely described in literature. A study by Jayaraman et al. ^12^ enrolled 108 patients whose CT angios were investigated for the incidence of internal jugular vein compression by extrinsic structures in the upper neck. They found 24.1% right side compressions and 30.6% left side compressions. They concluded that jugular vein compression is an anatomic variant and unlikely to be pathologic in nature. However, they did not investigate about the reasons why these CT angios were performed or patients’ clinical history [12].

We collected 8 cases of Eagle jugular syndrome. In our study the symptomatology is readily different respect the to the classic or the carotid syndrome, where ipsilateral pain was the more significant symptom (Table 1). Headache, numbness and dizziness are usually chronic in the jugular group and may be related to the impaired cerebral venous outflow. Subarachnoid hemorrhage usually presents with an acute headache (described as the worst headache ever) often associated to vomiting and altered consciousness. In few studies the compression of the IJV was related to pseudotumor cerebri [13, 14] or to headaches [13]. In many others, the jugular variant was significantly associated with persistent headache, and among comorbidities, to the peri-mesencephalic subarachnoid hemorrhage (p-SAH), both possibly related to cerebral venous hypertension [15, 16, 22, 23]. p-SAH is a particular kind of subarachnoid hemorrhage not associated to an evident vascular malformation, like aneurysms or arteriovenous malformations and with an usually good prognosis. The blood is mainly located in the cerebral basal cisterns and the cause is still debated. Among the hypothesized causes, a hypertensive venous bleeding (like coming from a particular variant of Basal vein of Rosenthal or an occluded venous sinus) is the most reliable [22, 23].

The relationship between the upper internal jugular vein bone nutcracker and the p-SAH could be a novel and intriguing finding. This variety of the subarachnoid hemorrhage is thought to be caused mainly by intracranial venous anomalies, but there are no reports in literature about its relationship with extracranial venous anomalies [22, 23]. In our series imaging at the intracranial level completely excluded the presence of any kind of vascular anomalies. However we hypothesized a jugular compression with a transient related venous hypertension could be a predisposing factor able to facilitate a p-SAH. On the basis of our study, if p-SAH should occur, we suggest extending also at the extracranial level the investigation of the brain vasculature, focusing particularly to the extracranial veins (Fig. 1). We also know that the compression of the upper segments of the internal jugular vein can induce chronic intracranial hypertension [24, 25], which was not found in our survey, yet.

CDU investigation allowed us to assess the venous haemodynamics in terms of flow velocity and flow rate of the internal jugular vein, especially just below the nutcracker, in the upper part of the neck. (Fig. 2) In the more severe cases the internal jugular vein appeared an empty vein, with no Doppler detectable flow in the lumen, even in favor of gravity with the head of the patients in upright position. Out of the initial assessment, CDU permits also to perform a precise and non-invasive follow-up of patients conservatively treated, in order to monitor any further flow worsening.

Alternatively, jugular outflow in case of external compression has been also extensively studied by the means of catheter venography protocol [26]. These authors, by the means of the same protocol, found out that balloon-angioplasty is not an effective treatment in case of external compression of IJV [27]. CT-angio in our patient clearly demonstrated an external compression by the styloid process. Considering that one of the main advantages of venography is the possibility to couple diagnostic and endovascular treatment (not indicated in our case, for the above-mentioned reasons) we decided to use CDU to measure the flow and open surgery to treat the obstruction.

In our experience, the treatment is usually conservative, especially when symptoms are not invalidating and apparently controlled by medical treatment. Only one patient underwent surgery and endovascular balloon angioplasty, with intriguing results (see illustrative case). In this case both headache and facial edema did not respond to conservative treatment. Indeed, Higgins et al. [15] stated that in their group of patients with idiopathic intracranial hypertension and impairment in venous outflow, most of those who benefited only did so after subsequent jugular stenting reinforced the surgical decompression. Moreover, Zhou et al. [28] reported in the same field their positive experience with exclusive jugular stenting in a group of 15 patients with IJV stenosis, but without bone compression.

Someone may argue that a potential indication could the prevention of recurrent episodes of cerebral hemorrhage. However, this is a rare eventuality following p-SAH (1/1220 as reported by Mensing et al. [22]).

The major limitation of our study is to be a retrospective analysis. Indeed, for the jugular variant we retrospectively enrolled 8 patients which are not enough to imply causation. Moreover we did not use a classification of the internal jugular vein phlebograms.

However, our findings are intriguing and warrant further investigations on the basis of a prospective multicenter epidemiologic data collection.

## Conclusions

Our results suggest a possible association of jugular impingement by an elongated styloid process with symptoms. In case of an elongated styloid process, venous narrowing should be always investigated, especially in patients presenting with sign and symptoms of intracranial hypertension and may represent in perspective a field of interdisciplinary interest.

## Data Availability

The datasets used and/or analyzed during the current study are available from the corresponding author on reasonable request.

## Abbreviations

CDU: Color doppler ultrasound
CSA: Cross sectional area
CT: Computer tomography
CTA: Computer tomography angiography
DUS: Doppler ultrasound
ENT: Ear nose and throat
IJV: Internal jugular vein
MRI: Magnetic resonance imaging
p-SAH: Peri-mesencephalic subarachnoid hemorrhage
TAV: Time-averaged maximum blood flow velocity
VA: Vertebral artery
VV: Vertebral vein
